# Limited Nrf2 activation and heterogeneous thyroidal effects in a 424-compound multi-assay screen call for rigorous testing of purported antioxidant and health-promoting supplements

**DOI:** 10.1016/j.redox.2026.104222

**Published:** 2026-05-16

**Authors:** Georgios Psarias, Panos G. Ziros, Athina Mageiropoulou, Dionysios V. Chartoumpekis, George I. Habeos, Basil Mohammed Alomair, Leonidas Duntas, Ioannis P. Trougakos, Gerasimos P. Sykiotis

**Affiliations:** a Service of Endocrinology, Diabetology and Metabolism, Lausanne University Hospital and University of Lausanne, Lausanne, 1011, Switzerland; b Division of Endocrinology, School of Medicine, University of Patras, Patras, 26500, Greece; c Department of Internal Medicine, College of Medicine, Jouf University, Aljouf, 04631, Kingdom of Saudi Arabia; d Evgenidion Hospital Unit of Endocrinology, Diabetes, and Metabolism, Thyroid Section, National and Kapodistrian University of Athens, Athens, Greece; e Department of Cell Biology and Biophysics, Faculty of Biology, National and Kapodistrian University of Athens, Athens, 15784, Greece

**Keywords:** Keap1-Nrf2 pathway, Thyroid redox homeostasis, Natural product library, Nutritional supplements, Thyroglobulin regulation, Sodium-iodide symporter

## Abstract

We screened 424 natural compounds, including many marketed as dietary supplements, to assess their antioxidant and thyroidal effects. In rat PCCL3 thyroid follicular cells, we performed parallel primary screens for antioxidant response element (ARE) activation, thyroglobulin (Tg) promoter activity, sodium/iodide symporter (NIS) promoter activity and iodide uptake. Hits were tested in downstream analyses, including ARE activation in Nrf2-knockout cells, an Nrf1 stabilization reporter, endogenous Nqo1, Tg and NIS mRNA and protein levels, and relationships between these readouts. Of 424 compounds, 69 induced ARE activity in wild-type cells; most also activated the ARE in Nrf2-knockout cells, and only 8 showed a profile consistent with predominantly Nrf2-dependent ARE activation. Several Nrf2-independent ARE inducers also stabilized an Nrf1-based reporter. Thirty compounds activated the Tg promoter and 26 significantly modulated iodide uptake, with only partial concordance between promoter activity, mRNA/protein expression and iodide uptake. Notable hits included genistein and apigenin, which showed balanced ARE and Tg induction together with NIS upregulation, supporting further evaluation in hypothyroidism and redifferentiation; luteolin, chrysin and diosmetin, which enhanced NIS expression and/or iodide uptake, highlighting potential as redifferentiation or adjunctive agents in thyroid cancer; emodin and gamma-oryzanol, which increased iodide uptake, suggesting utility in hypothyroidism or iodine deficiency; and bavachin, which showed the strongest iodide uptake induction without markedly increasing NIS protein, warranting mechanistic follow-up. Thyroid-relevant bioactivity was enriched among dietary supplements. Many compounds displayed complex Nrf2-dependent and -independent actions that can confer indication-specific thyroid benefits or risks, emphasizing the need for thyroid-focused evaluation of antioxidant and natural supplements.

## 1 Introduction

Natural compounds, particularly phytochemicals, are actively studied for their potential health benefits. Found in a variety of plant-based foods like fruits, vegetables, whole grains, and nuts, these bioactive nutrients are of interest for the prevention and/or treatment of chronic conditions such as metabolic, neurodegenerative and autoimmune diseases, and cancers [[1], [2], [3], [4], [5], [6], [7]]. Their accessibility as nutritional supplements makes them a practical addition to daily diet. One of the main mechanisms of action potentially leading to beneficial effects is via preventing or countering oxidative stress, a condition that results from an imbalance between oxidants and antioxidants causing damage to proteins, DNA, and lipids, and increasing the risk for various diseases [8]. However, other studies question the health benefits of various antioxidants, with some trials reporting limited to no efficacy, along with potential risks. This includes multiple clinical trials on antioxidant supplements aimed at cancer prevention that failed to demonstrate protective effects, highlighting concerns about using high-dose isolated compounds rather than whole foods for disease prevention [9]. For example, in studies of antioxidants like beta-carotene, supplementation has been linked with increased incidence of lung cancer and cardiovascular issues in high-risk groups, such as smokers and those exposed to asbestos [10].

Accumulating evidence links reactive oxygen species (ROS) generation and oxidative damage to the development of thyroid diseases, including thyroid cancer, autoimmune thyroiditis, and hyper- or hypothyroidism. Moreover, thyroid disorders themselves may trigger or worsen ROS production and oxidative stress, thereby amplifying oxidative damage [[11], [12], [13]]. In this context, nutritional therapy with antioxidant-rich foods and supplements has been proposed as complementary treatment against thyroid disorders [[14], [15], [16]]. On the other hand, the use of certain natural compounds has been reported to potentially cause thyroidal side-effects. For instance, certain flavonoids, commonly found in various plant-based foods, have shown potential inhibitory effects on thyroid hormone production by suppressing thyroid peroxidase (TPO) activity, which can lead to increased serum levels of thyroid-stimulating hormone (TSH) and goiter development in some cases [17]. Consequently, while antioxidant-rich foods and supplements are explored as preventive or therapeutic measures for thyroid health, carefully considering their potential side-effects is crucial in avoiding thyroid-related risks.

Dietary supplements are widely marketed over-the-counter for thyroid health and typically contain minerals, vitamins, and plant-derived bioactive compounds intended to support thyroid function, with recent studies suggesting that targeted micronutrient or antioxidant supplementation may confer measurable physiological or metabolic benefits in selected populations [18]. Several of these ingredients possess intrinsic antioxidant activity or enhance endogenous antioxidant defenses, which may be particularly relevant given the thyroid gland's physiological exposure to oxidative stress during hormone synthesis [19]. Minerals such as selenium [14] and zinc contribute indirectly to redox homeostasis as cofactors of antioxidant enzymes, while numerous botanical constituents, including polyphenols, flavonoids, and terpenoids, exert direct antioxidant effects or activate cellular antioxidant response pathways [17]. Importantly, some of these natural compounds have also been shown to modulate thyroid-specific processes, including thyroglobulin (Tg) expression and iodide handling [20]. However, antioxidant activity does not uniformly predict beneficial thyroid effects, as certain compounds like resveratrol may interfere with thyroid hormone synthesis or iodide uptake [21], underscoring the need for thyroid-specific evaluation of dietary supplements rather than extrapolation from general antioxidant properties.

Thyroid hormone synthesis is a complex process involving the ROS hydrogen peroxide (H_2_O_2_) that is crucial in initiating thyroid hormone production, which involves iodide oxidation and iodination of Tg, the precursor protein of thyroid hormones. To counterbalance this, the thyroid's antioxidant defense system must effectively regulate ROS scavenging [13]. Like in other tissues, the signaling pathway comprising nuclear factor erythroid 2-related transcription factor 2 (Nrf2) and its inhibitor Kelch-like ECH-associated protein 1 (Keap1) as its central players serves as a critical protective mechanism in the thyroid [22]. In addition to modulating antioxidant responses, Nrf2 also directly upregulates the production of Tg, through binding to antioxidant response elements (AREs) on regulatory regions of the Tg gene [19]. These connections between Nrf2 and thyroidal redox balance and hormone synthesis suggest that Nrf2 dysregulation could lead to thyroid dysregulation. Indeed, mice with reduced Keap1 expression and constitutive Nrf2 activation present diffuse goiter and increased TSH levels [20]; in humans, genetic activation of Nrf2 signaling in the thyroid has also been linked to goiter [23,24], and functional promoter polymorphisms in *NFE2L2* (the gene that encodes Nrf2) can predispose to auto-immune thyroiditis in conjunction with other genetic variations [25].

Several studies have assessed individual natural compounds for their effects on Nrf2 and/or the thyroid [20]. However, systematic studies simultaneously assessing collections of natural compounds for their thyroidal and Nrf2-mediated antioxidant effects are lacking. To address this knowledge gap, we conducted a cell-based screen of a library of 424 natural compounds, including several commonly used dietary supplements, across a panel of antioxidant- and thyroid-related assays; we report compounds with previously unknown effects on critical thyroidal aspects in Nrf2-dependent or -independent manners.

## 2 Methods

### 2.1 Compound library

**Fig. 1:**
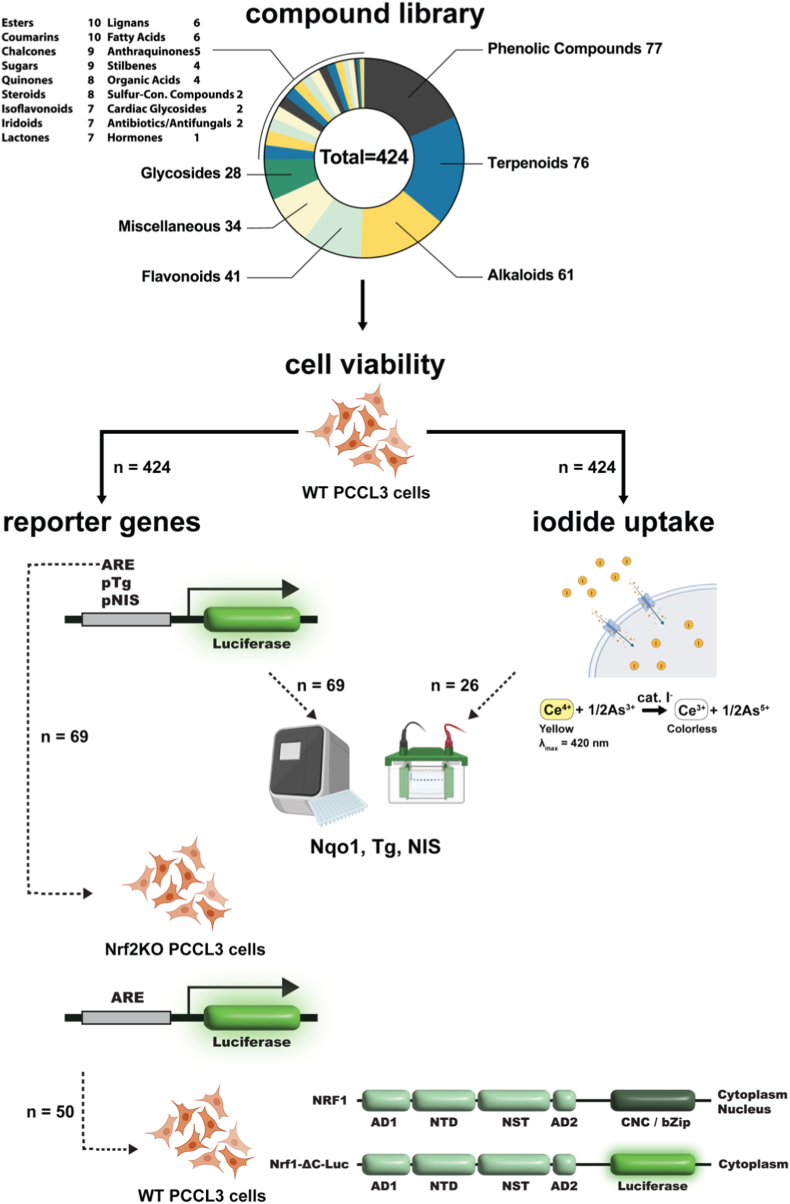
Workflow for the assessment of the collection of 424 compounds for their effects on thyroid function and antioxidant response pathways. Primary screens are indicated by solid arrows and were performed in wild-type (WT) PCCL3 cells. Starting with a cell viability assay (CellTiter-Glo® 3D Cell Viability Assay), all compounds were subsequently analyzed at selected non-toxic concentrations (Supplementary Table 1) using four further primary assays: three reporter gene activity assays (ARE-Luc, pTg-Luc, and pNIS-Luc) and an iodide uptake assay. Hits in these primary assays were further tested in downstream assays (indicated by dotted arrows), namely quantitative PCR and Western blot analyses for Nqo1, Tg, and NIS for all hits; ARE-Luc in Nrf2KO PCCL3 cells for hits in the ARE-Luc assay in WT cells; and Nrf1 luciferase fusion protein assay (Nrf1-ΔLuc) in WT PCCL3 cells for hits in the ARE-Luc assay in Nrf2KO PCCL3 cells.

A custom library (Cherry Pick Library) of 424 compounds was provided by Selleck Chemicals (Supplementary Table 1). The library was a subset of the Natural Product Library (Catalog No. L1400, Selleck Chemicals), from which antibiotics were excluded. The screen was performed manually using parallel cell-based assays. The workflow of the primary screen and downstream assays is shown in Fig. 1.

### 2.2 Cell culture

Rat PCCL3 cells were maintained as described previously [19] in Coon's modified Ham's F-12 medium supplemented with 5% fetal bovine serum (FBS), and a six-hormone (6H) mixture (consisting of TSH, insulin, apo-transferrin, hydrocortisone, somatostatin and glycyl-L-histidyl-l-lysine acetate). Experiments were conducted in medium containing 0.2% FBS and the six hormones (6H low-serum medium: reporter gene assays, real-time PCR and Western blots) or in low-serum medium without insulin (5H low-serum medium: iodide uptake assay).

### 2.3 Reporter gene assays

PCCL3 cell lines stably transfected with different constructs were generated in previous studies or as part of the present work, using standard protocols as previously detailed [19]. The following reporter constructs were used: (i) ARE-luciferase (ARE-Luc), bearing the ARE present in the 5′-flanking region of the rat NAD(P)H dehydrogenase 1 (Nqo1) gene [19,26,27]; (ii) Tg promoter-luciferase (pTg-Luc), bearing the upstream enhancer and the proximal basic promoter of human Tg [19,[28], [29], [30]]; (iii) Sodium (Na)/Iodide Symporter (NIS) promoter-luciferase (pNIS-Luc), bearing a 2.8 kb fragment of the rat NIS gene promoter [31]; and (iv) a protein-based reporter for NRF1 that lacks the DNA-binding (CNC/bZIP) domain fused to luciferase (named NRF1ΔC-Luc) [32]. Additionally, a PCCL3 Nrf2 knockout (Nrf2KO) cell line stably transfected with the ARE-Luc was used [19].

Each compound was tested on PCCL3 cells in 96-well plates with 8 technical replicates for 24 h under low-serum conditions at a concentration that was not toxic to the cells. To evaluate the toxicity of the compounds and to normalize the outcomes of the various reporter assays, cell viability was measured using the CellTiter-Glo® 3D Cell Viability Assay Kit (Promega, Madison, Wisconsin), which quantifies ATP levels as a proxy for the number of viable cells. Compounds showing reduced viability (<90%) at a concentration of 10 μM were further tested at lower concentrations (1 μM, 0.1 μM or 0.01 μM, until no toxicity was observed). Luciferase activities from various reporters described above were measured using the Luciferase Assay System (Promega, Madison, WI).

### 2.4 Iodide uptake assay

The iodide uptake assay was based on the methodology of Waltz et al. [33], with some modifications. PCCL3 cells seeded in 48-well plates were starved for 24 h in 5H medium and then treated for another 24 with compounds. Six technical replicates were used for each compound to account for small differences in cell numbers in each well as well as other random variability in the assay. At the end of the treatment, the medium was replaced with Hank's Balanced Salt Solution (HBSS) supplemented with 10 μM sodium iodide (NaI). In some wells of each plate, sodium perchlorate (100 μM) served as a negative control for unspecific iodide uptake. Following a 30 min incubation at 37 °C, the cells were washed 3 times with cold (4 °C) HBSS, and then 150 μL of 50% ethanol in water was added to each well. This modification fixes the cells in the wells while allowing iodide to be extracted from the cells. Fixed cells can be used to measure the DNA content, while a small portion of the extraction medium can be used to measure ATP content as a proxy of the number of cells as described above. At this point, plates can be securely sealed and stored at −20 °C, and the iodide assay can be continued later. Iodide measurement was performed in 96-well clear plates using 100 μL of the 50% ethanol extract and known iodide standards from 100 nM to 700 nM NaI in 50% ethanol. Iodide was determined by a spectrophotometric method based on the Sandell-Kolthoff reaction as described elsewhere with the modifications mentioned above [33].

### 2.5 RNA isolation and real-time PCR

After a 24-h low-serum period, cells seeded in 12-well plates were treated with selected compounds for 24 h. Total RNA was extracted using TRIzol reagent (Invitrogen) and further purified with NucleoSpin RNA Columns (Macherey-Nagel). For cDNA synthesis, 500 ng of RNA was used with the High-Capacity cDNA Reverse Transcription Kit (Applied Biosystems). The synthesized cDNA was diluted 40-fold, and 5 μL of this dilution was utilized in each 10 μL reaction volume for real-time PCR as previously described [19]. The housekeeping gene *Rpl19* served as reference.

### 2.6 Protein isolation and western immunoblotting

Protein extracts were obtained from PCCL3 cells seeded in 12-well plates using 200 μL of NuPAGE LDS Sample Buffer (#NP0008, Invitrogen) supplemented with a protease inhibitor cocktail (#78438, Thermo Scientific) and 50 mM DTT. Protein electrophoresis and Western blotting were carried out as described elsewhere [19]. Primary antibodies used included anti-thyroglobulin (#A0251, 1:5000 in 5% BSA in TBST, 3 h, Agilent), anti-rat NIS (#ABC1453 1:3000 in 5% BSA in TBST, overnight, Sigma-Aldrich), anti-NQO1 (#62262S, 1:4000 in 5% BSA in TBST, overnight, Cell Signaling Technology), and anti-β-Actin (#sc-47778HRP 1:3000 in EveryBlot Blocking Buffer, 1 h, Santa Cruz Biotechnology). Secondary antibodies were anti-mouse HRP and anti-rabbit HRP, both used at a dilution of 1:6000 (#7076S and #7074S, respectively, Cell Signaling Technology). β-actin served as a loading control; when protein values were quantified, they were normalized against β-actin.

## 3 Results

### 3.1 ARE-Luc primary screen in PCCL3 cells and counter-screen in Nrf2KO cells

**Fig. 2:**
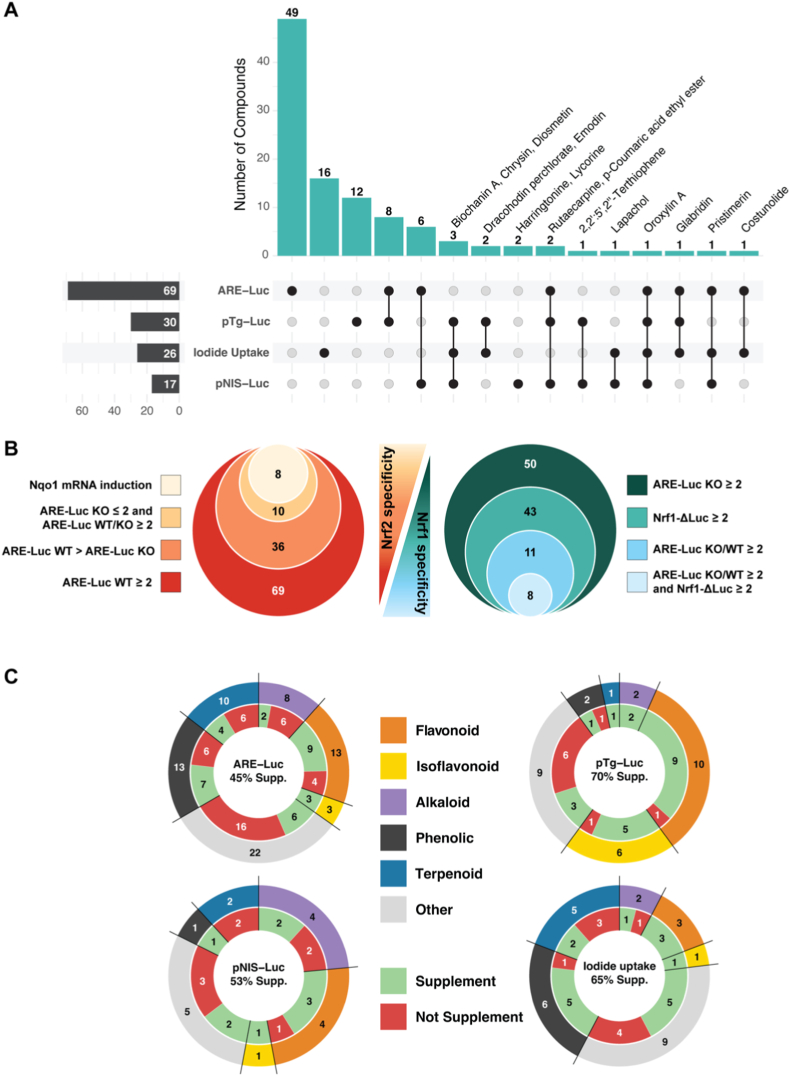
Comprehensive summary of multi-assay screening hits, Nrf2/Nrf1 specificity, and chemical enrichment. **(A)** UpSet-style matrix with accompanying bar plot displaying the number of hit compounds identified in each primary assay and the extent of their pairwise and higher-order overlaps. The full list of hits per assay is shown in Supplementary Table 2. **(B)** Nested diagrams summarizing Nrf2-and Nrf1-related specificity among ARE-Luc inducers in WT and Nrf2KO cells. **(C)** Chemical classification and dietary supplement enrichment. Donut charts display the distribution of major chemical classes (outer ring) and the proportion of dietary supplements (inner ring) for hits in each primary assay.

**Fig. 3:**
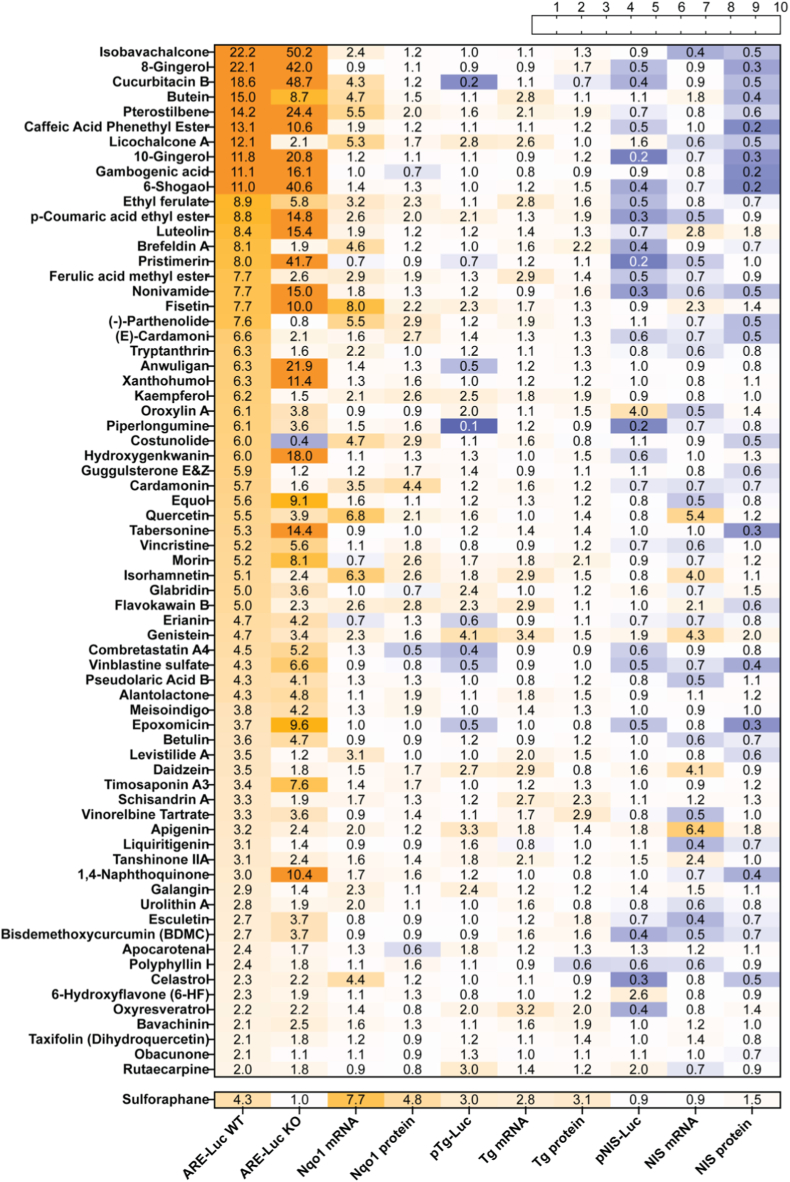
Heatmap displaying compounds that induced the ARE-Luc reporter ≥2-fold relative to control in WT PCCL3 cells (first column). The second column shows ARE-Luc reporter activity in Nrf2KO PCCL3 cells. The next columns show respective mRNA levels, protein levels and gene reporter activities in WT PCCL3 cells. Values represent fold-change relative to control. Sulforaphane serves as a positive control for ARE activation.

**Fig. 4:**
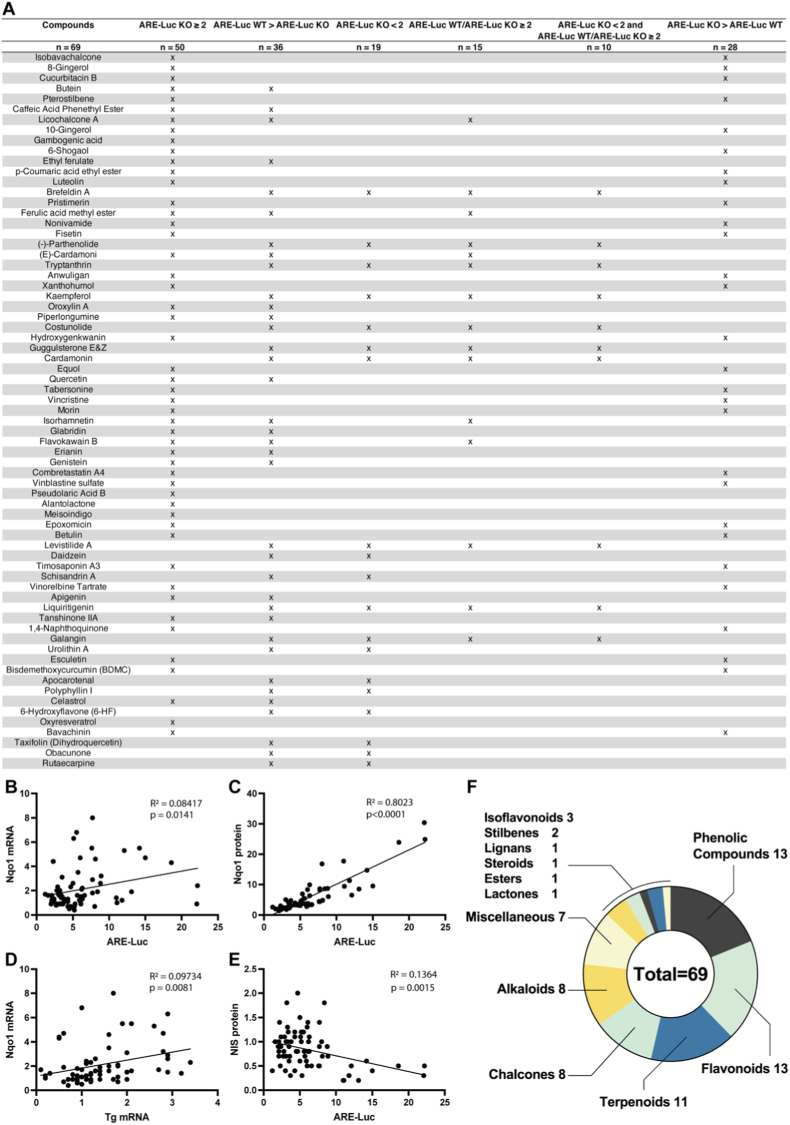
(A) Classification of compounds listed in Fig. 2 based on their ARE-Luc activity in WT and Nrf2KO PCCL3 cells. (B-E) Correlations between ARE-Luc activity and Nqo1 mRNA (B), ARE-Luc activity and Nqo1 protein expression (C), Tg mRNA and Nqo1 mRNA expression (D), and ARE-Luc activity and NIS protein expression (E). R^2^ values and p-values indicate the strength and statistical significance of correlations. (F) represents the number of compounds assigned to each chemical class from Fig. 2.

**Fig. 5:**
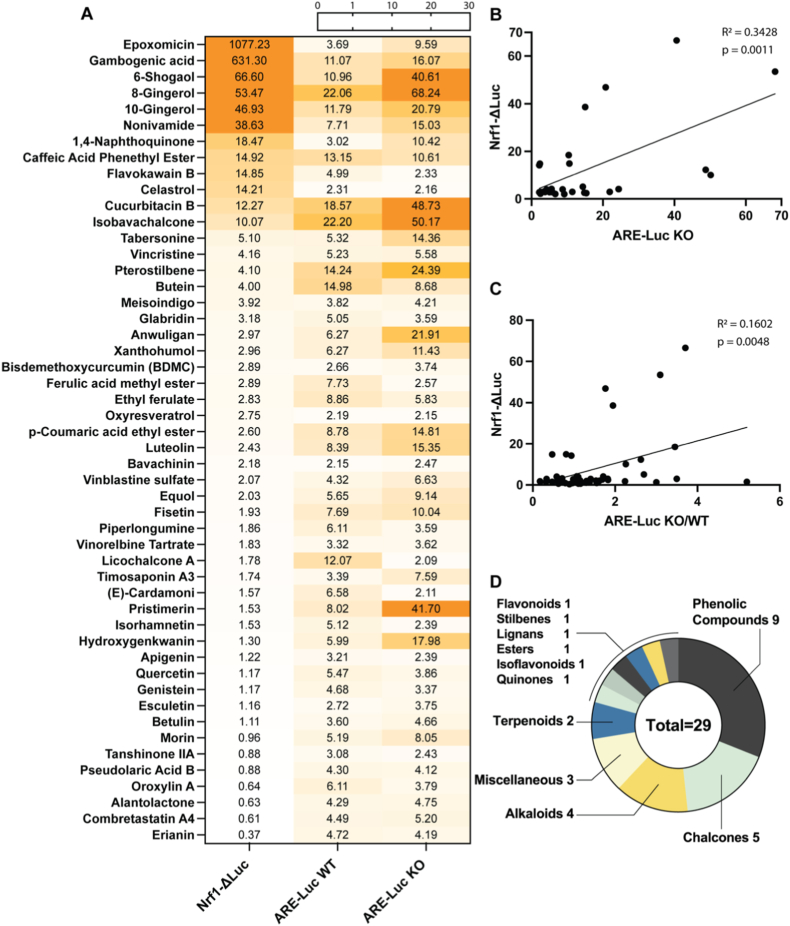
(A) Heatmap displaying compounds that induced the ARE-Luc reporter ≥2-fold in PCCL3 Nrf2KO cells. Data shown represent fold-change in Nrf1-ΔLuc in WT PCCL3 cells, and ARE-Luc in WT and Nrf2KO PCCL3 cells, relative to respective controls. (B) Correlation between Nrf1-ΔLuc activity in WT cells and ARE-Luc activity in KO cells. (C) Correlation between baseline Nrf1-ΔLuc activity and the relative ARE-Luc induction ratio (KO/WT). The extreme inducers Epoxomicin and Gambogenic acid were excluded from the correlation. (D) Chemical class distribution of the 29 compounds inducing ≥2-fold activation in the Nrf1-ΔLuc assay.

Of the 424 compounds screened, 50 (11.8%) exhibited toxicity in wild-type (WT) PCCL3 cells after 24-h exposure under low-serum conditions at 10 μM; these compounds were subsequently retested at progressively 10-fold lower concentrations until non-toxic concentrations were reached (Supplementary Table 1). Of the 424 compounds, 69 (16.2%) showed ≥2-fold induction in the ARE-Luc assay (Fig. 2A–B, Fig. 3, Supplementary Table 2); these were tested further in Nrf2KO cells. Interestingly, 50/69 compounds showed ≥2-fold ARE induction in Nrf2KO cells (Fig. 2B and. 4A); this is consistent with the fact that other factors can also bind and regulate the ARE [34], prompting us to further investigate Nrf2-independent mechanisms. Among the 69 compounds, 36 showed significantly higher activation in WT cells than in Nrf2KO cells, suggesting a central role of Nrf2 in mediating this response (Fig. 2B and. 4A). Of the 69 compounds, 19 induced ARE-Luc <2-fold in Nrf2KO cells, and 15 showed a ratio of ARE-Luc WT/ARE-Luc KO ≥ 2; combining these two criteria yielded only 10 compounds with a profile indicative of Nrf2-dependent ARE activation, matching the pattern associated with sulforaphane; for example, brefeldin A and parthenolide (Fig. 4A). In contrast, 28 compounds exhibited significantly higher ARE-Luc activity in Nrf2KO cells than in WT cells, suggesting a partially Nrf2-independent mechanism of action; for example, isobavachalcone and 8-gingerol (Fig. 4A). Additionally, of the 50 compounds that showed ≥2-fold ARE induction in Nrf2KO cells, 29 also stabilized a Nrf1 protein reporter by ≥ 2-fold (Fig. 5A); among these, epoxomicin showed the highest induction in the Nrf1 stabilization assay, consistent with its known mechanism as a specific proteasome inhibitor that (among other actions) activates Nrf1 [35]. Similarly, gambogenic acid, which induces endoplasmic reticulum (ER) stress, also highly stabilized the Nrf1 protein reporter, likely due to Nrf1's sensitivity to ER stress as part of the unfolded protein response (UPR) [36]. When these extreme inducers were excluded, there was a positive correlation between Nrf1 protein reporter induction (≥2-fold) and ARE-Luc activity (≥2-fold) in Nrf2KO cells (Fig. 5B), and with ratio of ARE-Luc activity in Nrf2KO vs. WT cells (Fig. 5C).

### 3.2 Correlations of ARE-Luc with antioxidant and thyroidal genes

All 69 ARE-Luc-inducing compounds were also tested for effects on the mRNA and protein levels of Nqo1, Tg and NIS, and on pTg-Luc and pNIS-Luc activity (Figs. 1, 2A and. 3). Nqo1 mRNA levels were upregulated ≥2-fold by 24 compounds (Fig. 3), and there was a positive correlation between ARE-Luc activity and Nqo1 mRNA levels and protein expression (Fig. 4B–C). Further, Nqo1 mRNA levels correlated positively with Tg mRNA levels (Fig. 4D) and ARE-Luc activity correlated negatively with NIS protein expression (Fig. 4E). No correlations were observed between ARE-Luc activity and NIS mRNA, Tg mRNA or pTg-Luc activity.

Among the 69 compounds inducing ARE-Luc activity at non-toxic concentrations in WT cells, the predominant chemical classes were phenolic compounds (n = 13, 18.8%), flavonoids (n = 13, 18.8%), and terpenoids (n = 10, 14.5%) (Fig. 2C and. 4F, Supplementary Table 1). Chi-square analysis showed significant a ∼2-fold enrichment of flavonoids in the ARE-inducing subset compared to the total library of 424 compounds (Fig. 2C) (18.8% vs. 9.7%, χ^2^ = 7.9, df = 1, p = 0.0048). In contrast, phenolic compounds (χ^2^ = 0.03, df = 1, p = 0.8725) and terpenoids (χ^2^ = 0.54, df = 1, p = 0.4632) did not deviate from their expected proportions (18.2% and 17.9%, respectively). Notably, some compounds, such as 8-gingerol (phenolic), enhanced ARE-Luc activity without elevating Nqo1 mRNA or protein levels. Similarly, pristimerin (a terpenoid) induced ARE-Luc activity in Nrf2KO cells, suggesting potential Nrf2-independent mechanisms.

From the 29 compounds that upregulated ARE-Luc activity in Nrf2KO cells and Nrf1-ΔLuc activity in WT cells by ≥ 2-fold at non-toxic concentrations, the predominant chemical classes were phenolic compounds (n = 9, 31.0%), chalcones (n = 5, 17.2%), and alkaloids (n = 4, 13.8%). Chi-square analysis revealed a highly significant 8-fold enrichment of chalcones compared to the total library (17.2% vs. 2.1%, χ^2^ = 34.6, df = 1, p < 0.0001), whereas phenolic compounds (χ^2^ = 3.5, df = 1, p = 0.0627) and alkaloids (χ^2^ = 0.009, df = 1, p = 0.9257) did not show significant enrichment (Fig. 2C and. 5D).

### 3.3 Human tg promoter primary screen

**Fig. 6:**
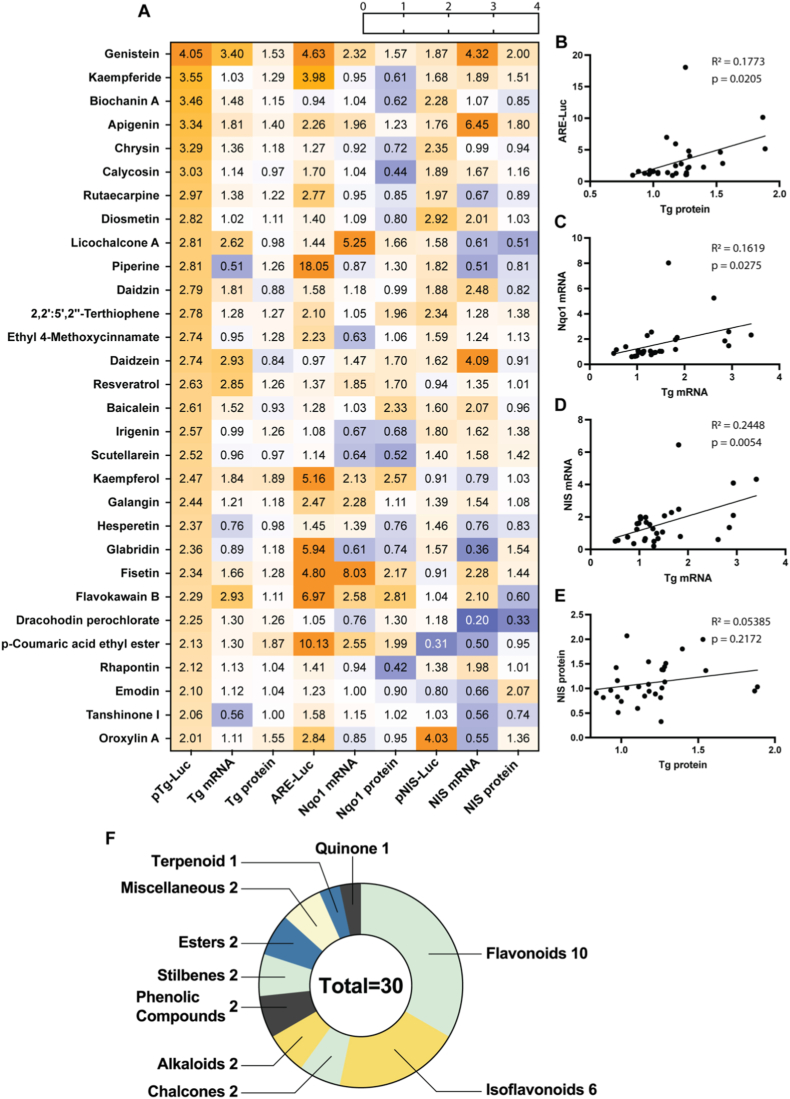
(A) Heatmap displaying compounds that induced the pTg-Luc reporter assay ≥2-fold in WT PCCL3 cells (first column). The following columns show respective mRNA and protein levels for Tg, Nqo1, NIS and ARE-Luc activity. Values represent fold-change relative to control. (B-E) Correlation between Tg protein levels and ARE-Luc reporter activity (B) Tg mRNA and Nqo1 mRNA levels (C), NIS mRNA and Tg mRNA levels (D) and NIS protein and Tg protein levels (E). (F) represents the number of compounds assigned to each chemical class from panel (A).

Of the 424 compounds screened, 30 induced Tg promoter activity by ≥ 2-fold (Fig. 2A and. 6A, Supplementary Table 2). For these 30 compounds, mRNA and protein levels of Tg, Nqo1 and NIS, and NIS-Luc reporter activity were quantified. A positive correlation was observed between ARE-Luc activity and Tg protein levels (Fig. 6B), as well as between Nqo1 and Tg mRNA levels (Fig. 6C). Additionally, a positive correlation was found between Tg and NIS mRNA levels (Fig. 6D) but not protein levels (Fig. 6E); what these two genes have in common is their translational upregulation by the TSH signaling pathway [37,38]. On the other hand, NIS mRNA and protein levels showed no correlation with either ARE-Luc activity or Nqo1 levels, which indicates that NIS is not regulated by Nrf2 signaling, consistent with prior *in vivo* data [19,22]. Overall, these findings demonstrate that compounds activating the Nrf2 pathway can upregulate Tg expression without directly influencing NIS regulation.

The 30 compounds inducing the Tg promoter reporter were predominantly flavonoids (n = 10, 33.3%) and isoflavonoids (n = 6, 20.0%). Chi-square analysis revealed significant enrichment of these classes relative to the full compound library, with flavonoids being 3.5-fold more represented (33.3% vs. 9.7%; χ^2^ = 20.7, df = 1, p < 0.0001), and isoflavonoids 12.1-fold more represented (20.0% vs. 1.65%; χ^2^ = 66.3, df = 1, p < 0.0001) (Fig. 2C and. 6F).

### 3.4 Iodide uptake and NIS promoter activity primary screens

In the pNIS-Luc assay, only 8 of 424 compounds induced ≥2-fold promoter activation, of which only 2 increased NIS mRNA levels by ≥ 2-fold, while none enhanced NIS protein expression by ≥ 1.5-fold (Fig. 2A, Supplementary Fig. 1A, Supplementary Table 2). Conversely, 9 compounds reduced pNIS-Luc activity to ≤0.5-fold; all were associated with decreased NIS mRNA levels, and 5 of these also reduced NIS protein expression, although the magnitude of repression varied. While pNIS-Luc activity showed statistically significant correlations with both NIS mRNA and protein expression across all compounds for which data were available from downstream assays (Supplementary Fig. 1B–C), fold changes in pNIS-Luc, mRNA, and protein levels were often not concordant on a compound-by-compound basis (Supplementary Fig. 1A<). Together, these data indicate that although NIS promoter activity contributes to regulation of NIS expression, it does not reliably predict the extent of mRNA or protein modulation, supporting the involvement of post-transcriptional and/or post-translational regulatory mechanisms.

**Fig. 7:**
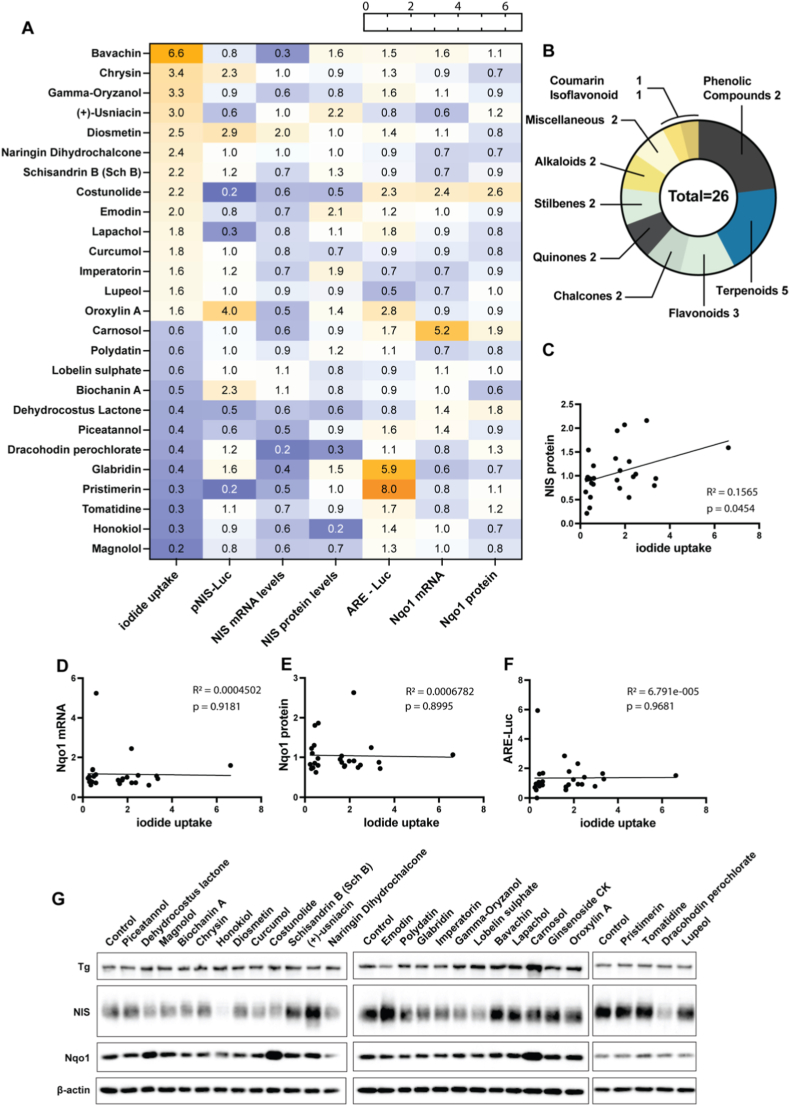
(A) Heatmap displaying compounds that increased iodide uptake ≥1.5-fold or decreased it ≤0.6-fold (first column). The following columns show respective pNIS-Luc activity, NIS mRNA and protein levels, ARE-Luc activity and Nqo1 mRNA levels. (B) Represents the number of compounds assigned to each chemical class from panel (A). (C–F) Correlations between iodide uptake and NIS protein (C), Nqo1 mRNA (D), Nqo1 protein (E) and ARE-Luc activity (F). (E) Representative western blots showing the expression of Tg, NIS, and Nqo1 proteins in WT PCCL3 cells following treatment with selected compounds.

Among the 424 compounds screened, 26 exhibited a significant effect on iodide uptake, with either an increase of ≥1.5-fold or a decrease of ≤0.6-fold relative to control; for these compounds, the mRNA and protein levels of Tg, NIS, and Nqo1 were also measured (Fig. 7A and G). Overall, among the 14 compounds that increased iodide uptake, only 7 also increased NIS protein levels, of which only 3 increased pNIS-Luc activity and none increased NIS mRNA levels. Among the 12 compounds that decreased iodide uptake, 9 also decreased NIS protein levels, of which only 4 decreased pNIS-Luc activity; the same 4 compounds also decreased NIS mRNA levels. Thus, the concordance between effects on iodide uptake and NIS-pLuc, NIS mRNA levels and NIS protein levels is overall low, though potentially somewhat better for iodide uptake-decreasing compounds. Nevertheless, a positive correlation was observed between iodide uptake and NIS protein expression (Fig. 7C). For example, emodin both enhanced NIS protein levels and increased iodide uptake by ≥ 2-fold, indicating a direct regulatory effect on NIS, though not at the transcriptional level. On the other hand, other compounds, such as gamma-oryzanol, increased iodide uptake without increasing NIS mRNA or protein levels, implying a mechanism independent of direct NIS upregulation. Finally, there was no correlation between iodide uptake and either Nqo1 mRNA or protein or ARE-Luc activity (Fig. 7D–F). Interestingly, some compounds with strong antioxidant activity, such as carnosol, which is promoted online as a booster of “thyroid health”, exhibited inhibitory effects on NIS expression and iodide uptake, suggesting that, despite their antioxidant potential, these compounds may negatively impact thyroid function.

### 3.5 Enrichment of dietary supplements among bioactive compounds

To determine whether thyroid-relevant bioactivity, including thyroglobulin expression and iodide uptake, or ARE-inducing capacity is associated with dietary supplement availability, all compounds in the library were screened for their classification as dietary supplements (i.e., marketed preparations of single compounds, multiple compounds or extracts from natural sources associated with specific compounds). Among 424 screened compounds, 69 were classified as ARE-inducing, of which 31 (44.9%) were dietary supplements, compared with 94/355 non-ARE-inducing compounds (26.5%). This difference was significant (χ^2^ = 9.54, df = 1, p ~ 0.002; φ = 0.15), indicating enrichment of supplements among ARE-inducing compounds (Figs. 2C–ure 1 and 4F).

Compounds inducing pTg-Luc showed an even stronger enrichment: of 30 pTg-Luc-inducing compounds, 21 (70%) were dietary supplements, compared with 105/394 non-inducing compounds (26.6%) (χ^2^ = 22.74, df = 1, p < 0.00001; φ = 0.23), demonstrating a significant enrichment of supplements among compounds activating Tg transcription (Figs. 2C–ure 1 and 6F).

Similarly, dietary supplements comprised 78.6% (11/14) of iodide uptake stimulators and 50.0% (6/12) of suppressors, compared with 27.6% of the 398 compounds with no effect. This difference was significant across groups (χ^2^ = 7.30, df = 2, p = 0.0002), with post-hoc analyses showinga that enrichment was driven by iodide uptake stimulators, which were significantly more likely to be supplements than no-effect compounds (χ^2^ = 16.03, df = 1, p = 0.0001), whereas uptake suppressors did not differ significantly. Collectively, these findings indicate that compounds exhibiting functional activity in ARE activation, Tg promoter induction, and iodide uptake enhancement are significantly enriched among dietary supplements (Figs. 2C–ure 1 and 7B).

## 4 Discussion

### 4.1 Nrf2-dependent and -independent ARE inducers

The 69 compounds identified to activate the ARE (Fig. 3) comprised a diverse set with varying therapeutic potential according to the literature. When the 10 compounds that met criteria favoring Nrf2-mediated ARE induction (ARE-Luc KO < 2 and ARE-Luc WT/KO ≥ 2, Fig. 4A) were further filtered by Nqo1 mRNA induction ≥2, only 8 compounds remained: parthenolide, costunolide, cardamonin, levistilide A, kaempferol, galangin, tryptanthrin and brefeldin A. In contrast to the other 7 compounds, which are generally known Nrf2 activators, brefeldin A, which showed the most potent ARE induction, is an inducer of ER stress that has been shown to activate the Nrf2 pathway indirectly through the accumulation of misfolded proteins and subsequent oxidative stress [39]. In thyroid cancer cells, miR-1284 functions as a tumor suppressor by inhibiting proliferation and migration while promoting apoptosis, and its exosomal release is markedly reduced by Brefeldin A treatment [40]. Parthenolide upregulated ARE-Luc and Nqo1 mRNA levels similarly to sulforaphane, indicating its potency as an Nrf2 activator in thyroid cells. It was previously reported to induce HMOX1 (HO-1) and GCLM in human thyroid cancer (BCPAP) cells [41], suggesting thyroidal activation of Nrf2. Like parthenolide, costunolide is a α,β‐unsaturated sesquiterpene lactone; it robustly triggers Nrf2/ARE signaling [42]. In addition to activating Nrf2, it markedly increased iodide uptake (2.2-fold) without influencing NIS expression. The flavonoids kaempferol and galangin are also established Nrf2 activators: kaempferol markedly promotes NRF2 nuclear translocation and HO-1 induction in macrophages [43], and galangin upregulates hepatic Nrf2/HO-1 and other antioxidant defenses [44]. Albeit a general antioxidant, kaempferol was found to decrease iodide retention in a thyroid cancer cell line by increasing iodide efflux [45]; in the present study, however, we did not observe a significant effect on iodide uptake in WT cells. Cardamonin (a chalcone) was shown to activate Nrf2 and raise HO-1/NQO1 in chondrocytes [46], and levistilide A induced ferroptosis via NRF2/HO-1 activation in breast cancer cells [47]. Likewise, tryptanthrin engaged the Keap1/Nrf2 signaling pathway in a murine ulcerative colitis model, where it alleviated oxidative stress and inflammation [48]. In conclusion, brefeldin A, parthenolide, costunolide and kaempferol have been reported to exert effects in benign or malignant thyroid cells, suggesting that their potential thyroidal impact should be carefully considered when used as supplements or therapeutic agents.

Several of the compounds we identified were able to upregulate ARE-driven reporter activity even in Nrf2KO cells, indicating that their mechanism of action involves at least partial Nrf2-independence. Flavonoids such as quercetin, genistein, fisetin, luteolin and apigenin exemplify this profile, exhibiting moderate ARE activation likely mediated through both Nrf2-dependent and alternative transcriptional pathways. Compared to more potent inducers like chalcones and gingerols, these flavonoids show a distinct pharmacological behavior, coupling modest antioxidant signaling with effects on thyroid-specific gene expression. Notably, they upregulate the transcription of NIS, suggesting engagement of transcriptional networks beyond oxidative stress regulation. This dual functionality distinguishes flavonoids from ARE activators that focus solely on redox modulation. The ability to enhance NIS expression is particularly relevant in contexts of iodine deficiency or endocrine dysfunction, where improved iodide uptake could support hormone production. These findings highlight the translational potential of compounds with mixed Nrf2-dependent and -independent mechanisms, especially for targeting conditions involving both oxidative and thyroid-related pathologies.

ARE activity is commonly used as a primary readout in screens for Nrf2 inducers [49]. However, the fact that only 8/424 total natural compounds and 8/69 ARE activators seemed Nrf2-specific cautions against relying excessively on ARE activity for this purpose and favors complementary or alternative approaches like assays for inhibition of the Nrf2-Keap1 interaction for compounds that bind to the Kelch domain of Keap1 [50], assays for betaTrCP-mediated Nrf2 regulation as occurs under low-serum conditions [51], as well as secondary assays in Nrf2KO cells as done herein. The present findings suggest that, contrary to what is commonly held, truly Nrf2-specific natural inducers may be relatively limited. Further, while Nrf2 induction is often reported in studies on the aforementioned flavonoids to support the involvement of Nrf2 in the observed *in vitro* or *in vivo* effects, the actual dependency on Nrf2-mediated mechanisms is rarely examined [52]. The present findings highlight the importance of systematically considering the potential Nrf2-independent activities of such compounds.

### 4.2 New potential Nrf1 activators

Interestingly, compounds traditionally not associated with Nrf2 activation were found to induce the ARE, including gambogenic acid, vincristine, vinblastine sulfate, and vinorelbine tartrate. These agents are primarily recognized for their anticancer properties through mechanisms like ferroptosis induction or microtubule disruption [53,54]. In our screen, they demonstrated moderate to strong ARE induction in WT cells, i.e., 11.1-, 5.2-, 4.3-, and 3.3-fold for gambogenic acid, vincristine, vinblastine sulfate and vinorelbine tartrate, respectively. Notably, this activation was preserved in Nrf2KO cells, with ARE-Luc induction values of 16.1-, 5.6-, 6.6-, and 3.6-fold, respectively. Regarding gambogenic acid, while related compounds like gambogic acid have been linked to Nrf2 activation in models of inflammation, gambogenic acid itself is not documented to directly upregulate ARE/Nrf2; rather, it typically targets ferroptosis pathways such as miR-1291/FOXA2 or AMPKα/SLC7A11/GPX4 in colorectal and lung cancers [[55], [56], [57], [58]]. Similarly, the vinca alkaloids vincristine, vinblastine sulfate, and vinorelbine tartrate are established chemotherapeutics that inhibit mitosis by binding tubulin but are not known to activate ARE/Nrf2. In fact, high Nrf2 levels often confer resistance to these drugs, as seen in B-cell acute lymphoblastic leukemia, where Nrf2 overexpression reduces vincristine sensitivity through PI3K-AKT modulation; similarly, in non-small cell lung cancer, the Nrf2/p-Fyn/ABCB1 axis drives vinorelbine resistance without evidence of drug-induced Nrf2 upregulation [59,60]. The comparable (if not higher) ARE induction by these compounds in Nrf2KO cells compared to WT cells indicates Nrf2-independent mechanisms. One such potential mechanism is the activation of the related cap'n’collar transcription family member Nrf1, which, like Nrf2, binds AREs as a dimer with small Maf proteins [61]. Despite their similar DNA-binding mechanisms, Nrf1 and Nrf2 fulfill distinct biological roles and regulate sets of target genes that overlap only partially [62]. We therefore examined whether compounds upregulating the ARE in Nrf2KO cells showed activation of Nrf1.

Indeed, when we tested Nrf2-independent ARE inducers in the Nrf1 stabilization assay (Nrf1-ΔLuc), we observed induction by several compounds. These included vincristine, vinblastine sulfate and vinorelbine tartrate, which induced mild induction (4.2-, 2.1-, and 1.8-fold, respectively), and gambogenic acid, which induced strong activation (631.3-fold). Gambogenic acid is a natural xanthone that has been shown to induce ER stress [63] in tumor cells through ROS production and to trigger the UPR in human prostate cancer cells [64]. Regarding vincristine, several studies report that it triggers ER stress and activates UPR pathways. In rat models of chemotherapy-induced neuropathy, vincristine markedly upregulated canonical UPR markers (PERK, IRE1α, ATF6) and the ER chaperone GRP78/BiP; this was accompanied by increased pro-apoptotic Bax and caspase-3 and decreased Bcl-2, consistent with ER stress associated apoptotic signaling [65]. Vinblastine also engages ER stress pathways in cancer cells, and vinblastine treatment of breast cancer cells upregulates GRP78/BiP, induces XBP1 mRNA splicing and increases eIF2α phosphorylation, which are hallmarks of UPR activation [66]. These findings are particularly relevant given the established role of ER stress and the UPR in regulating Nrf1 activation, as Nrf1 is anchored in the ER membrane and is believed to undergo stress-dependent processing that facilitates its transcriptional activation [67].

As expected, known proteasome inhibitors like epoxomicin and celastrol were also found to also stabilize Nrf1-ΔLuc [68,69]. For other compounds, the mechanisms are less clear but warrant further investigation. For example, ginger-derived pungent compounds, namely gingerols such as 8-gingerol (53.5-fold) and 10-gingerol (46.9-fold), and their dehydrated analog 6-shogaol (66.6-fold), and capsaicinoids like nonivamide (38.6-fold), form a cluster of mild electrophilic stressors. These agents induce moderate oxidative and proteotoxic stress, thereby upregulating cytoprotective pathways without overt cytotoxicity. For example, 6-shogaol and nonivamide are reported to trigger both antioxidant and heat shock protein responses, indicating a mild proteostasis disruption [70,71] that could account for Nrf1 activation. Another mechanistic class encompasses quinone-based stressors like 1,4-naphthoquinone and the triterpene celastrol. These compounds undergo redox cycling to generate ROS and electrophiles that impose significant proteotoxic stress. Quinones such as 1,4-naphthoquinone are well-known activators of Nrf2-mediated antioxidant gene expression via Keap1 thiol modification, helping cells counter oxidative damage. Celastrol, also a quinone, directly suppresses proteasome activity at higher concentrations, causing misfolded protein accumulation [68]; at such proteasome-inhibiting doses, celastrol markedly increases Nrf1 processing and nuclear translocation, similar to classical proteasome inhibitors [68]. Thus, quinone-based compounds that functionally impair the proteasome likely provoke redox stress and engage the Nrf1-mediated proteasome recovery pathway.

Our assessment for Nrf1 activation was based on the Nrf1-ΔLuc reporter assay. When we assayed genes that are reportedly regulated specifically by Nrf1 (as opposed to Nrf2) in other tissues, the effects observed were less consistent. This variability may stem from cell-type-specific factors, as studies have shown that tissue-specific cofactors or modulatory proteins may alter Nrf1's ability to engage specific promoters effectively [61], and that cellular stress pathways can lead to differential Nrf1 stabilization and activation patterns across tissue types [72]. Further work is warranted to characterize the identified compounds regarding their Nrf1-inducing properties and mechanisms, such as the induction of Nrf1-regulated proteasome subunits that are ubiquitously expressed.

The identification of pharmacological activators of Nrf1 is of particular importance, as Nrf1 has emerged as a central regulator of cellular proteostasis and stress adaptation, yet remains far less explored than Nrf2. Unlike Nrf2, whose pharmacological activation has already translated into approved therapies, Nrf1 activation has been historically difficult to target due to its complex ER-associated regulation and reliance on stress-dependent processing mechanisms [73]. Nevertheless, recent screening campaigns demonstrate that Nrf1 is a tractable target and can be engaged by chemically diverse small molecules. Notably, high-throughput and targeted screens have identified compounds capable of enhancing Nrf1-dependent transcription through distinct upstream perturbations, including proteotoxic stress, ER stress, and mechanisms independent of direct proteasome inhibition. For example, Laconelli et al. showed that small molecules can selectively stimulate Nrf1-driven transcriptional programs, revealing both proteasome-linked and proteasome-independent routes to Nrf1 activation [74]. More recently, Sedláček et al. identified non-toxic Nrf1 activators that enhance proteasome biogenesis and proteostasis without inducing overt cellular stress, highlighting the therapeutic potential of selectively boosting the Nrf1 pathway [75]. Together, these studies support the concept that Nrf1 can be pharmacologically activated via multiple mechanistic entry points and reinforce the relevance of identifying novel Nrf1 modulators, particularly in disease contexts characterized by chronic proteotoxic, ER, or oxidative stress – such as thyroid cancer [76] and thyroid orbitopathy [77] – where restoring adaptive stress responses may provide substantial benefit.

Regarding compounds that activated the ARE-Luc reporter to greater magnitude in Nrf2-KO than in Nrf2-WT cells some were active in the Nrf1 assay (e.g., 6-shogaol, 8-gingerol, cucurbitacin B, xanthohumol), but others were not (e.g., pristimerin and hydroxygenkwanin). For the latter compounds, various mechanisms may be involved. Electrophilic compounds like pristimerin may generate higher intracellular ROS levels in the absence of Nrf2; this heightened oxidative stress could trigger compensatory activation of alternative ARE-binding transcription factors such as Nrf3 [78,79]. Additionally, for both electrophilic and non-electrophilic compounds, as well as for Nrf1-activating compounds, the absence of Nrf2 may relieve competitive inhibition at ARE sites, allowing increased binding by these alternative CNC-bZIP factors and removal of Bach1/Bach2 repression under oxidative conditions [80].

### 4.3 ARE-dependent and -independent thyroglobulin regulation

In addition to serving as the backbone for thyroid hormone synthesis in normal thyroid, Tg is also used as a serum marker during the follow-up of patients with differentiated thyroid carcinoma (DTC). We have previously shown that Tg is directly upregulated by Nrf2 via two functional AREs [19], and that human genetic variation in Keap1/Nrf2 signaling correlates with circulating Tg levels [81]. The positive correlations observed here between ARE-Luc activity and Tg protein levels (Fig. 6B), and between Nqo1 and Tg mRNA levels (Fig. 6C) are also consistent with Tg being a target gene of Nrf2. Notwithstanding these overall correlations, the relative induction of ARE and Tg-related readouts was variable among compounds. On the one hand, flavonoids such as genistein and apigenin displayed balanced induction of ARE-Luc assay and Tg-related readouts. Genistein (4.1-fold pTg-Luc, 4.6-fold ARE-Luc, 3.4-fold ARE-Luc KO), an isoflavone available in supplements, has been shown to improve thyroid hormone levels and reduce levels of TSH and autoantibodies in patients with Hashimoto's thyroiditis, potentially via Nrf2-mediated antioxidant effects in the thyroid [82,83]. However, high doses may affect thyroid homeostasis, highlighting dose-dependent risks [84]. Apigenin (3.3-fold pTg-Luc, 1.8 pNIS-Luc, 6.5-fold NIS mRNA, 2.3 ARE-Luc, 2.4-fold ARE-Luc KO) stands out for increasing NIS transcription, consistent with preclinical evidence that, in combination with Akt inhibition, it enhances NIS expression and iodide uptake, thereby counteracting TGFβ-mediated NIS suppression in PCCL3/Tet-On BRAF^V600E^ cells (a RAI-R DTC model) [85,86].

On the other hand, compounds like resveratrol (2.6-fold pTg-Luc, 1.4-fold ARE-Luc) and daidzein (2.7-fold pTg-Luc, 1.0-fold ARE-Luc) that showed weaker ARE-Luc activation relative to pTg induction have been reported as thyroid disruptors. Resveratrol can act as a goitrogen and interfere with hormone homeostasis, potentially exacerbating hypothyroidism in susceptible individuals [21]. Daidzein, similar to genistein, modulates thyroid hormone levels and may induce microfollicular changes, underscoring the goitrogenic potential of isoflavones at higher exposures [87]. In contrast, fisetin (2.3-fold pTg-Luc, 4.8-fold ARE-Luc, 10-fold ARE-Luc KO) and kaempferol (2.5-fold pTg-Luc, 5.1-fold ARE-Luc, 1.5-fold ARE-Luc KO) exhibited stronger ARE activation relative to pTg induction with only minor effects on NIS, supporting their antioxidant roles without marked thyroidal interference. Interestingly, kaempferol enhances energy expenditure and thyroid hormone activation, suggesting benefits for metabolic disorders [88,89]. Finally, other compounds in our panel, such as biochanin A (3.5-fold pTg-Luc, 0.9-fold ARE-Luc) and licochalcone A (2.8-fold pTg-Luc, 1.4-fold ARE-Luc), exhibited modest Tg induction with weak or negligible ARE activation; biochanin A, in particular, inhibited proliferation in thyroid cancer cells through cell cycle arrest and apoptosis, suggesting a potential therapeutic relevance in oncology [20], independently of NIS (1.1-fold mRNA). Overall, the varied responses discussed above underscore the compound- and context-specific roles, necessitating targeted studies to combine potential Nrf2-mediated benefits with thyroidal benefits, or balance the former against the risk of thyroid perturbations, depending on the case.

### 4.4 Functional modulation of iodide uptake by natural compounds

Effects on iodide uptake are of particular importance for translational application in various thyroid diseases, such as increasing iodide uptake in hypothyroidism, suppressing it in hyperthyroidism, and reactivating it in RAIR-DTC (redifferentiation) [90,91]. Even though iodide uptake is mediated by NIS, both literature [92,93] and the present findings (Fig. 7) show that compounds’ effects on iodide uptake are not always mirrored by respective changes in NIS expression, and, conversely, changes in NIS expression do not always translate into the expected effects on iodide uptake. We therefore discuss separately compounds that increased or decreased iodide uptake, or modulated NIS without affecting iodide uptake.

Bavachin exhibited the highest increase in iodide uptake (6.6-fold), which was apparently independent of Nrf2 activation, given its modest effects on ARE-Luc (1.5-fold) and Nqo1 mRNA (1.6-fold). It also suppressed NIS transcription (pNIS-Luc 0.8-fold, NIS mRNA 0.3-fold) and only modestly increased NIS protein levels (1.6-fold). Bavachin is primarily investigated in preclinical models for anti-inflammatory and neuroprotective properties [94] but lacks evidence from clinical trials or commercial availability as a supplement. Gamma-Oryzanol also demonstrated increased iodide uptake (3.3-fold). In the literature, administration of gamma-oryzanol has been reported to reduce elevated serum TSH levels in patients with primary hypothyroidism, an effect that appears to be mediated through a central hypothalamic mechanism involving modulation of monoaminergic pathways rather than through alterations in circulating thyroid hormone concentrations [95]. As a widely available supplement derived from rice bran oil, it has been evaluated in clinical trials for cholesterol management and anxiety, with emerging evidence suggesting that Nrf2 activation contributes to its antioxidant and neuroprotective properties [96,97]. The naturally occurring flavonoids chrysin and diosmetin enhanced iodide uptake (3.4- and 2.5-fold, respectively) and upregulated pNIS-Luc (2.3- and 2.9-fold, respectively), but only diosmetin increased NIS mRNA levels (1.0-fold and 2.0-fold, respectively) and none increased NIS protein levels (0.9-fold and 1.0-fold, respectively). Chrysin has been shown to activate Notch1 signaling in anaplastic thyroid carcinoma (ATC) cells, suppressing tumor growth and inducing apoptosis [98,99]. Similarly, diosmetin exhibits pro-apoptotic effects in ATC cells, likely through increasing oxidative stress and blocking the PI3K/Akt/mTOR pathway [100]. Both compounds also influence the Nrf2/ARE pathway in a context-dependent manner: they activate Nrf2 signaling and upregulate cytoprotective genes (e.g., HO-1, NQO1) in normal cells, thereby guarding against oxidative damage [101,102]. Conversely, they tend to inhibit Nrf2 activity in cancer cells, with chrysin potently suppressing Nrf2 in chemoresistant HeLa-derived BEL-7402/ADM cells (originally misidentified as a hepatocellular carcinoma line) via PI3K/Akt and ERK pathway inhibition and sensitizing to ROS-mediated apoptosis [103]. Finally, oroxylin A showed divergent effects on pNIS-Luc activity (4.0-fold), NIS mRNA levels (0.5-fold) and NIS protein levels (1.4-fold), with moderate effects on iodide uptake (1.6-fold). Available in extracts like Sabroxy® for cognitive support, oroxylin A has shown promise in human studies for memory enhancement but remains understudied regarding thyroidal effects [104].

The most potent suppressors of iodide uptake were the natural Magnolia bark compounds magnolol and honokiol (Fig. 7A), reducing uptake to 0.2- and 0.3-fold of control levels, respectively. They also suppressed pNIS-Luc activity and NIS mRNA and protein levels, suggesting that the suppressed iodide uptake is due to decreased NIS expression. Both compounds are bioactive biphenyl neolignans known for broad pharmacological effects that include anti-inflammatory and anticancer activities [105,106], but they have not been previously linked to iodide metabolism. The other two compounds that simultaneously suppressed iodide uptake and the three NIS-related read-outs were piceatannol and dehydrocostus lactone (DHL). Piceatannol is a naturally occurring stilbene polyphenol, structurally similar to resveratrol, found in grapes, berries, and passionfruit. It exhibits potent antioxidant effects by scavenging free radicals like hydroxyl and superoxide, often surpassing resveratrol due to its additional hydroxyl group, which enhances radical stabilization and cellular protection; is has therefore been tested as a supplement in preclinical studies and small human studies for metabolic and anti-aging benefits [[107], [108], [109]]. DHL, a sesquiterpene from Saussurea costus, shows antioxidant activity by scavenging ROS, thereby mimicking endogenous antioxidants [110,111]; it lacks human data and is not used as a commercial supplement. The remaining 8 compounds, which reduced iodide uptake without consistent effects on NIS-related readouts, are also of interest, because some of them might be combined with NIS-decreasing compounds to further suppress iodide intake. Among them, potent suppression of iodide uptake was shown by tomatidine (0.3-fold) and glabridin (0.4-fold). Tomatidine is a steroidal alkaloid aglycone found in green tomato fruits (*Solanum lycopersicum*) [112], while glabridin is a prenylated isoflavone from licorice root (*Glycyrrhiza glabra*) [113]. Both compounds exhibit antioxidant activity through direct ROS scavenging and upregulation of endogenous antioxidant enzymes [114,115]. Neither compound shows documented thyroidal effects. Tomatidine is not commercially available as an oral supplement, while glabridin exists primarily in topical formulations as a tyrosinase inhibitor for skin-lightning applications [113].

### 4.5 Compounds that modulate NIS without effects on iodide uptake

Finally, compounds that consistently modulated NIS-related readouts without affecting iodide uptake are also of potential interest for combined use with iodide uptake-modulating compounds. In particular, among the 9 compounds that downregulated pNIS-Luc activity, 6 also produced concordant reductions in NIS mRNA and protein levels, indicating coherent transcriptional repression (Supplementary Fig. 1**)**. Two of these compounds, bisdemethoxycurcumin (BDMC) and piperlongumine, are notable for their availability and use as dietary supplements or nutraceutical derivatives. BDMC, a naturally occurring curcuminoid and minor constituent of turmeric, has been reported to exert potent regulatory effects on redox-sensitive transcriptional pathways, including NF-κB and Nrf2 signaling [116]. Similarly, piperlongumine, an alkaloid derived from *Piper longum* and commonly marketed for its antioxidant and anti-inflammatory properties, demonstrated coordinated suppression of NIS promoter activity, mRNA and protein abundance. Mechanistically, piperlongumine is known to elevate intracellular oxidative stress selectively in certain cellular contexts and to perturb proteostasis and stress-response pathways [117], which may secondarily impair thyroid-specific gene expression. Harringtonine, a cephalotaxine alkaloid originally isolated from Cephalotaxus species, also emerged as a consistent down-regulator of NIS-related readouts, suppressing pNIS-Luc activity in parallel with reductions in NIS mRNA and protein levels, without directly affecting iodide uptake. Harringtonine is a well-characterized inhibitor of translation initiation, acting by blocking ribosomal A-site function on the 60S subunit and rapidly attenuating global protein synthesis [118]. Beyond its canonical role in translational inhibition, harringtonine has been shown to induce cellular stress responses secondary to proteostatic imbalance [119]. On the other hand, among the 8 compounds that upregulated pNIS-Luc activity, none produced concordant inductions in NIS mRNA and protein levels (Supplementary Fig. 1).

From a translational perspective, combinations of compounds that upregulate iodine uptake without affecting NIS with compounds that upregulate NIS could have additive or synergistic effects in the redifferentiation of RAIR-DTC. Supplementary Table 3 lists known natural compounds with previously demonstrated redifferentiation potential; among the 4 compounds included in our library, 3 (apigenin, genistein and luteolin) increased NIS mRNA and protein in PCCL3 cells, consistent with what has been previously shown in thyroid cancer cells [[120], [121], [122], [123]]. However, other compounds, such as resveratrol [[124], [125], [126], [127]], are known to have discordant effects between WT cells and cancer cells. These observations suggest that compounds upregulating NIS mRNA and protein in WT cells are worth testing in cancer cells even if they show no effect on iodide uptake in WT cells, and that combinations between iodine uptake-inducing and NIS-inducing compounds also warrant consideration on a one-by-one basis in cancer cells.

### 4.6 Conclusions and implications for the use of dietary supplements

Collectively, our findings underscore the need for proactive consideration of the effects of dietary supplements on thyroid function, as our screen revealed that many natural compounds modulated key thyroid-related and antioxidant pathways in unpredictable ways. Notably, several supplements widely marketed as antioxidants or “health enhancers” showed suppressive actions on thyroid markers. Thus, the natural origin or antioxidants properties of a compound do not safeguard against unanticipated thyroid effects, including potential suppression of iodide uptake or thyroid hormone synthesis under certain conditions. Given these complexities, we urge caution in the use of such supplements, especially among individuals with thyroid disorders or those receiving thyroid-targeted therapies, where unexpected modulation of NIS, Tg or iodide uptake could be detrimental. For example, NIS downregulation in DTC cells in a patient about to receive RAI could lead to treatment failure and cancer persistence, and Tg up- or downregulation in a patient with DTC during follow-up could lead to erroneous conclusions about disease progression or remission, respectively.

Considerations around use of Nrf2 activation by supplements are different compared to synthetic triterpenoids like omaveloxolone, which have been engineered to achieve sustained target engagement at nM concentrations [128]. In human supplementation studies with broccoli sprout or seed extracts, daily sulforaphane intakes in the range of currently marketed supplements typically yield transient peak plasma concentrations of sulforaphane and its conjugated metabolites in the sub-to low-μM range, which is in line with earlier pharmacokinetic work reporting total sulforaphane-metabolite peaks in the low-μM range after broccoli seed preparations [129]. In our screen, sulforaphane was therefore used as a positive control at 10 μM, and the remaining test compounds were screened at similar micromolar concentrations to allow a direct comparison with this established benchmark; however, without human pharmacokinetic data for most of these structures, their *in vitro* potency should be interpreted as a first filter for activity rather than a direct prediction of effective oral doses.

Due to the lack of physiologically relevant human thyroid follicular cell lines, the study used the PCCL3 rat thyroid cell line [130]; the two-dimensional culture precluded assaying for hormone synthesis/secretion, which would require the three-dimensional structures of thyroid follicles. Each compound was tested functionally at a single concentration, and secondary assays analyzed a limited gene panel; e.g., antioxidant activity was inferred from Nqo1 induction, without direct assays of redox status or oxidative stress. Notwithstanding these limitations, the focused screen of natural compounds in a well-characterized thyroidal model yields tissue-specific relevance, considering that most prior antioxidant screens did not use thyroid‐specific models [131]. Another strength is the comprehensive documentation of multiple primary and secondary readouts including ARE induction, functional cell viability as a dose-finding criterion, and Nrf1-and thyroid-related parameters. The integrated phenotypic and mechanistic data provided by these assays can be relevant for translational studies, especially because many of the active hits are common in foods or dietary supplements. Future work could include dose-response studies for compounds of potential therapeutic interest; combinations of compounds in preclinical models (e.g., a compound that induces iodide uptake without affecting NIS with a compound that increases NIS, to test for further iodide uptake induction); and *in vivo* pilot studies in cellular or mouse models of thyroid physiology or disease, such as redifferentiation of RAIR-DTC cells or protection from oxidative stress in models of hyper- or hypothyroidism or thyroid autoimmunity [132,133].

## CRediT authorship contribution statement

**Georgios Psarias:** Conceptualization, Visualization, Writing – original draft, Writing – review & editing. **Panos G. Ziros:** Conceptualization, Investigation, Supervision, Writing – original draft, Writing – review & editing. **Athina Mageiropoulou:** Investigation, Writing – review & editing. **Dionysios V. Chartoumpekis:** Investigation, Writing – review & editing. **George I. Habeos:** Investigation, Writing – review & editing. **Basil Mohammed Alomair:** Investigation, Writing – review & editing. **Leonidas Duntas:** Investigation, Writing – review & editing. **Ioannis P. Trougakos:** Investigation, Writing – review & editing. **Gerasimos P. Sykiotis:** Conceptualization, Funding acquisition, Methodology, Project administration, Supervision, Validation, Writing – original draft, Writing – review & editing.

## Declaration of competing interest

The authors declare that they have no known competing financial interests or personal relationships that could have appeared to influence the work reported in this paper.

## Data Availability

All data are included with the manuscript.
