# Supplementary figures and images for: Limited Nrf2 activation and heterogeneous thyroidal effects in a 424-compound multi-assay screen call for rigorous testing of purported antioxidant and health-promoting supplements
Source: Redox Biol. 2026 May 16;94:104222. doi: 10.1016/j.redox.2026.104222 (PMC13213695; doi:10.1016/j.redox.2026.104222)

*
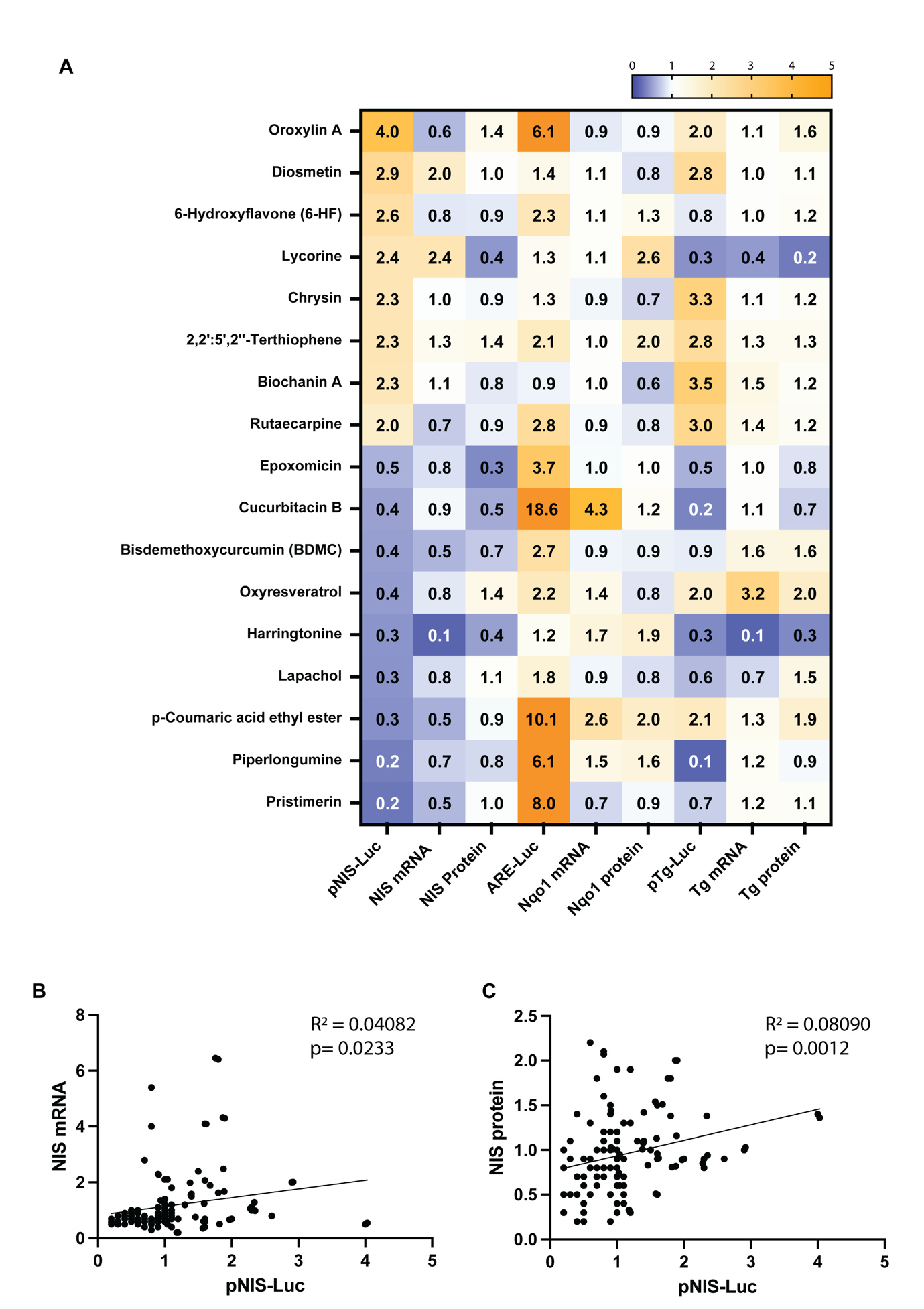
*

*Supplementary Figure 1*

Supplement: Multimedia component 2 [file mmc2.docx]
